# Severe Spotted Fever Group Rickettsiosis, Australia

**DOI:** 10.3201/eid1311.070099

**Published:** 2007-11

**Authors:** William J.H. McBride, Joshua P. Hanson, Robert Miller, Drew Wenck

**Affiliations:** * James Cook University, Cairns, Queensland, Australia; † Wellcome Trust-Mahidol University-Oxford Tropical Medicine Research Programme, Bangkok, Thailand; ‡ The Townsville Hospital, Douglas, Queensland, Australia; § Cairns Base Hospital, Cairns, Queensland, Australia

**Keywords:** Renal insufficiency, Rickettsia infection, Ixodes, purpura fulminans, pneumonia, Queensland, Australia, dispatch

## Abstract

We report 3 cases of spotted fever group rickettsial infection (presumed Queensland tick typhus) in residents of northern Queensland, Australia, who had unusually severe clinical manifestations. Complications included renal failure, purpura fulminans, and severe pneumonia. Clinical illness caused by *Rickettsia australis* may not be as benign as previously described.

Queensland tick typhus (QTT) is caused by *Rickettsia australis*, an obligate intracellular organism that is transmitted to humans through the bite of 1 of 2 known tick vectors, *Ixodes holocyclus* or *I*. *tasmani* (*1*). The disease occurs along the eastern coast of Australia, including Queensland. QTT is considered to be a mild illness. Clinical features are fever, headache, and myalgia. An eschar may be seen at the site of the tick bite, and a maculopapular or vesicular skin rash is usually noted. Patients usually make an uncomplicated recovery. We report 3 cases of presumed QTT in Australia that were unusually severe and showed manifestations that, to our knowledge, were previously unreported with this disease.

## The Cases

### Case 1

The first case occurred in a 40-year-old woman from Kuranda, Queensland, with a 1-week history of fever, myalgia, headache, and dry cough. She had sustained tick bites to the back and leg while planting trees. She was febrile (temperature 38.1°C). A widespread maculopapular rash with a minor petechial component was present. No eschars were found. Renal dysfunction was evident by blood tests (Table). A provisional diagnosis of leptospirosis was made, and she was treated with intravenous penicillin. Urea and creatinine levels continued to increase over 3 days to 14.8 mmol/L and 300 μmol/L, respectively, despite administration of intravenous fluids. She showed no dehydration or hypotension. Urinalysis showed 1+ proteinuria only. The patient’s clinical condition improved slowly over 7 days. The rash had a more noticeable petechial component at this stage of the disease. The patient was discharged on day 7 and given a 5-day course of oral doxycycline, 100 mg, twice a day. She had fully recovered on subsequent follow-up. A diagnosis of QTT was made retrospectively on the basis of serologic results..

**Table:** Laboratory test results in 3 severe cases of infection with *Rickettsia australis*, Australia

Test	Case 1	Case 2	Case 3	Reference range
Hemoglobin, g/L	124	130	142	115–160
Neutrophil count, cells/L	6.19	11.4	8.8	2.00–8.00 × 10^9^
Lymphocyte count, cells/L	0.39	0.4	1.58	1.00–4.00 × 10^9^
Platelet count, cells/L	63	30	167	140–400 × 10^9^
Sodium, mmol/L	131	135	139	135–145
Urea, mmol/L	9.8	27.8	5.0	3.0–8.0
Creatinine, μmol/L	190	430	100	70–120
Bilirubin, μmol/L	28	105	41	<20
Alkaline phosphatase, U/L	295	250	587	30–120
Gamma-glutamyl transferase, U/L	205	121	877	<50
Alanine aminotransferase, U/L	131	47	404	<40
Aspartate aminotransferase, U/L	178	152	417	<35
Leptospirosis immunoglobulin M	Negative	Negative	Negative	
Duration of symptoms before doxycycline treatment, d	17	14	10	
*R. australis* antibody titer*	Negative to 2,048 over 9 d	64–1,024 over 8 d	Negative to >1,024 over 9 d	Negative <64 Positive >64

*By immunofluorescence.

### Case 2

The second case occurred in a 69-year-old woman from Innisfail, Queensland, who was hospitalized with a 2-week history of fever, myalgia, neck pain, and confusion. She was febrile (temperature 39.2°C) and had tachycardia (140 beats/min). Multiorgan failure, purpura fulminans, and digital necrosis developed over a 2-day period, and she was transferred to an intensive care unit. Intubation and ventilation were required. She had widespread cutaneous and digital necrosis. There were no eschars or lymphadenopathy. Prothrombin time was 22 s (normal range 8–14 s), activated partial thromboplastin time was 53 s (normal range 25–38 s), and fibrinogen level was 1.6 g/L (normal range 1.5–4.0 g/L). The latex D dimer titer was 8 (normal <1). Results of a PCR for *Neisseria meningitidis* in blood and an extensive screen for primary vasculitides and prothrombotic disorders were negative. A skin biopsy specimen showed vessel thrombosis and no evidence of vasculitis, which is consistent with purpura fulminans.

**Figure 1:**
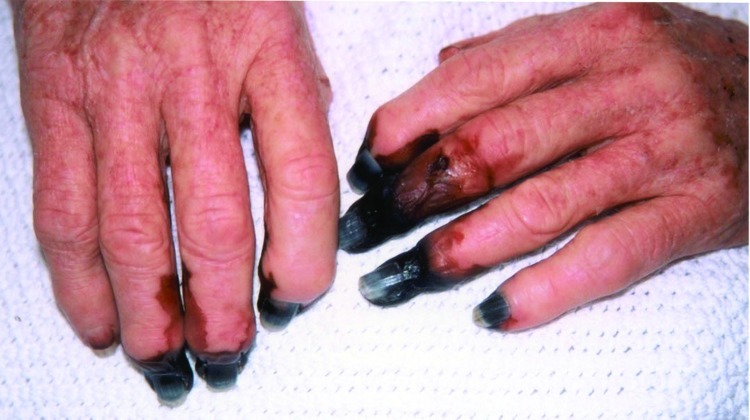
Digital gangrene in a patient (case 2) with *Rickettsia australis* infection.

Treatment included broad-spectrum antimicrobial drugs and doxycycline. Clinical recovery was prolonged, and she required temporary renal dialysis. Fourteen digital amputations were performed on her hands and feet (Figure 1). There was serologic evidence of *R*. *australis* infection. She lived in a house that bordered bushland and had received tick bites. However, she could no recall her last exposure. Tissue from the skin biopsy specimen was tested by PCR with primers against the rickettsial 17-kDa gene (*2*), and immunohistochemical analysis was performed with polyclonal rabbit antisera against spotted fever group (SFG) rickettsiae. Both tests showed negative results.

### Case 3

**Figure 2:**
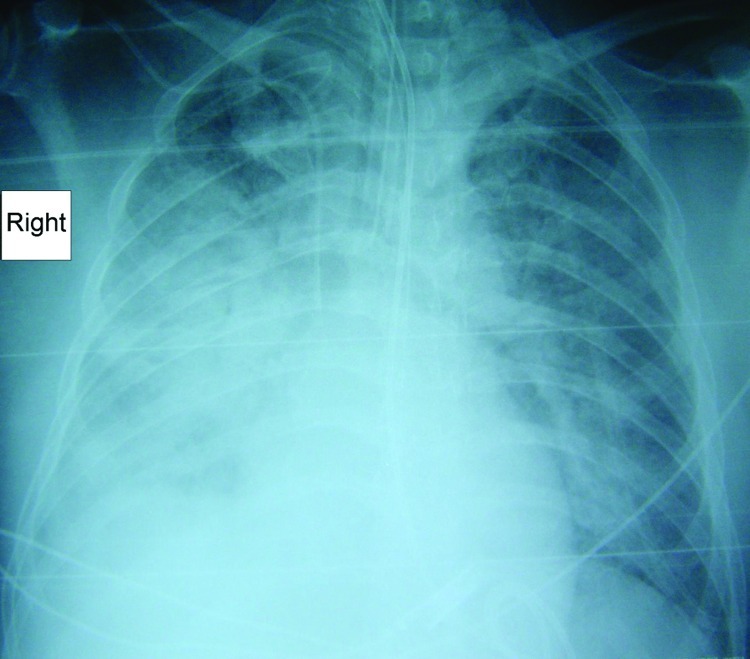
Radiograph showing pneumonia in a patient (case 3) with *Rickettsia australis* infection.

The third case occurred in a 45-year-old man from Deeral, Queensland, who was seen with headache, malaise, and vomiting 10 days after a tick bite. He also had a cough, dyspnea, and insulin-dependent diabetes. He was employed working on rural roads. He was febrile (temperature 38.2°C) and had tachycardia (145 beats/min) and tachypnea (40 breaths/min). He required 15 L/min oxygen by mask to maintain oxygenation. There was a fine petechial rash and left inguinal lymphadenopathy. A chest radiograph showed bilateral interstitial infiltrates (Figure 2). Over the ensuing hours, respiratory failure developed, and he required intubation and ventilation. Twelve hours after admission, his arterial blood gas results were pH 7.32, pO_2_ 59 mmHg, and pCO_2_ 55 mmHg on 100% oxygen while ventilated. His creatinine level increased to 180 μmol/L over the first 2 days and then slowly returned to normal. He was given broad-spectrum antimicrobial drugs, including doxycycline, on the day of admission. Treatment with doxycycline continued for 7 days. His recovery was marked by gross generalized edema. Serologic results were diagnostic for infection with *R*. *australis*. He was ventilated for 16 days and spent 3 weeks in the intensive care unit. He returned to work 2 months after admission.

## Conclusions

The clinical features of QTT have been described in 2 reviews (*1**,**3*). One review of 62 cases included patients from Flinders Island in the Bass Strait, an area now known to be endemic for a new rickettsia in the SFG, *Rickettsia honei (**4**)*. These reviews describe an illness marked by malaise, headache, and myalgia. A maculopapular rash appears in most patients. The rash may become petechial or vesicular in some cases. An eschar is seen in up to half the cases and lymphadenopathy in <70%. Less common clinical manifestations include joint pain, splenomegaly, cough, conjunctivitis, sore throat, nausea, abdominal pain, and photophobia. One patient had minor renal dysfunction (*1*). Mild-to-moderate elevation of hepatic transaminase levels is common.

One fatal case of QTT has been described in a 68-year-old man from northern Queensland (*5*). His illness was clinically marked by progressive renal failure, bilateral pulmonary infiltrates, acidosis, abnormal liver function test results, thrombocytopenia, and hypoprothrombinemia.

The first patient described in this report had moderate renal impairment, which is commonly associated with leptospirosis. Her renal dysfunction did not improve with rehydration but had fully resolved when her condition was reevaluated 3 weeks after hospital discharge. Renal failure is a feature of other spotted fever rickettsial illnesses. Rocky Mountain spotted fever (RMSF), which is caused by *R*. *rickettsii,* is associated with a multifocal perivascular interstitial nephritis. Renal dysfunction is believed to be a consequence of hypovolemia secondary to rickettsial disease–induced alterations in capillary permeability (*6**,**7*). Renal failure has also been recorded as a complication of infection with *R*. *conorii* (Mediterranean spotted fever) (*8*).

The second patient had a clinical picture usually associated with overwhelming bacterial infection. *N*. *meningitidis* was considered a possible cause of her illness. However, her 2-week illness before deterioration was not typical. Serologic results were diagnostic for infection with *R*. *australis*. Purpura fulminans has been described in RMSF. In a review of cases of gangrene complicating this infection, 6 cases with remarkable similarities to our second case were described (*9*).

The predominant clinical complication of the third patient was pulmonary involvement. This characteristic has only been described once in QTT (*5*). Pulmonary involvement has been described in RMSF, in which the pathology changes are thought to be related to noncardiogenic pulmonary edema consequent to capillary endothelial damage (*10**,**11*).

There are 12 described rickettsiae of the spotted group (*12*). RMSF is associated with a mortality rate of 7%, even with treatment (*5*), and *R*. *conorii* has been associated with severe disease and fatal cases. Other rickettsiae are considered to cause mild illness.

The 3 cases described here were seen over a 4-year period at Cairns Base Hospital in northern Queensland. We are aware of 2 other cases seen at other hospitals, 1 complicated by renal failure, confusion, abnormal coagulation test results, and impaired gas exchange, and the other with severe pneumonia (P. Marshall, R. Miller, pers. comm.).

Although QTT is a mild disease in most patients, its diagnosis should now be considered in patients who reside in or visit the rickettsial disease–endemic area of eastern coastal Australia and are hospitalized with renal failure or impaired pulmonary function. Delays in seeking treatment may have contributed to illness severity in 2 of our patients. Given the serologic cross-reactivity between members of the SFG rickettsia, it cannot be assumed that all cases described were caused by *R*. *australis*. Another rickettsia of the SFG has been recently described in northern Queensland (*13*), and distinguishing between species will be important in future studies.
